# Costing the supply chain for delivery of ACT and RDTs in the public sector in Benin and Kenya

**DOI:** 10.1186/s12936-014-0530-1

**Published:** 2015-02-05

**Authors:** Rima Shretta, Brittany Johnson, Lisa Smith, Seydou Doumbia, Don de Savigny, Ravi Anupindi, Prashant Yadav

**Affiliations:** Systems for Improved Access to Pharmaceuticals and Services (SIAPS), Management Sciences for Health (MSH), 4301 North Fairfax Drive, Suite 400, Arlington, VA 22203 USA; Global Health Group, University of California, San Francisco, 550 16th Street, 3rd Floor, San Francisco, CA 94158 USA; Swiss Tropical and Public Health Institute, Socinstrasse 57, PO Box 4002, Basel, Switzerland; University of Basel, Petersplatz 1, 4001 Basel, Switzerland; William Davidson Institute (WDI), University of Michigan, 724 East University Avenue, Ann Arbor, MI 48109 USA; Ross School of Business, University of Michigan, 701 Tappan Street, Ann Arbor, MI 48109 USA; School of Public Health, University of Michigan, Ann Arbor, MI 48109 USA

**Keywords:** Malaria, Costs, ACT, RDTs, Supply chain, Public sector, Distribution, Delivery

## Abstract

**Background:**

Studies have shown that supply chain costs are a significant proportion of total programme costs. Nevertheless, the costs of delivering specific products are poorly understood and ballpark estimates are often used to inadequately plan for the budgetary implications of supply chain expenses. The purpose of this research was to estimate the country level costs of the public sector supply chain for artemisinin-based combination therapy (ACT) and rapid diagnostic tests (RDTs) from the central to the peripheral levels in Benin and Kenya.

**Methods:**

A micro-costing approach was used and primary data on the various cost components of the supply chain was collected at the central, intermediate, and facility levels between September and November 2013. Information sources included central warehouse databases, health facility records, transport schedules, and expenditure reports. Data from document reviews and semi-structured interviews were used to identify cost inputs and estimate actual costs. Sampling was purposive to isolate key variables of interest. Survey guides were developed and administered electronically. Data were extracted into Microsoft Excel®, and the supply chain cost per unit of ACT and RDT distributed by function and level of system was calculated.

**Results:**

In Benin, supply chain costs added USD 0.2011 to the initial acquisition cost of ACT and USD 0.3375 to RDTs (normalized to USD 1). In Kenya, they added USD 0.2443 to the acquisition cost of ACT and USD 0.1895 to RDTs (normalized to USD 1). Total supply chain costs accounted for more than 30% of the initial acquisition cost of the products in some cases and these costs were highly sensitive to product volumes. The major cost drivers were found to be labour, transport, and utilities with health facilities carrying the majority of the cost per unit of product.

**Conclusions:**

Accurate cost estimates are needed to ensure adequate resources are available for supply chain activities. Product volumes should be considered when costing supply chain functions rather than dollar value. Further work is needed to develop extrapolative costing models that can be applied at country level without extensive micro-costing exercises. This will allow other countries to generate more accurate estimates in the future.

**Electronic supplementary material:**

The online version of this article (doi:10.1186/s12936-014-0530-1) contains supplementary material, which is available to authorized users.

## Background

In recent years, pricing of medicines and health commodities has become more transparent and readily available through resources including the International Drug Price Indicator Guide and the Global Fund Price and Quality Reporting mechanism [1,2]. Although this transparency has allowed for more accurate cost estimates, supply chain costs for delivering specific products—are still poorly understood and undefined [3,4]. Previous studies demonstrated that supply chain costs account for a significant proportion of total programme costs [5-7]. Surveys have found that when measured as a percentage of the total acquisition cost, supply chain costs ranged from 1% for contraceptives in Bangladesh to 13% for essential health commodities in Ghana [8], 18% for ACT in Malawi and up to 44% for large-volume bed nets in Liberia [9]. Such “ballpark” estimates frequently determine the budget presented to donors [10] and are often insufficient for delivering the products to health facilities. Author analysis of Global Fund proposals during Rounds 8–10 found that between 3 and 14% of total procurement costs are earmarked for procurement and supply chain management (PSM) functions in Africa [11]. Given that donors are placing a greater emphasis on value for money and that commodities consist about a quarter of all health care costs [12], using accurate supply chain cost estimates is key to improving access to medicines and health commodities [13].

Estimating the supply chain costs of a public health system is complex for several reasons. Most public health supply chains, particularly those in low- and middle-income countries, do not systematically track supply chain costs and rarely keep records on expenditure by each supply chain function. Additionally, multiple stakeholders including donors, government agencies, and international organizations are responsible for different aspects of the supply chain. Finally, the supply chain costs are often integrated within other costs and capturing the costs from each stakeholder, particularly indirect costs, is an onerous task. Anti-malarial medicines are often distributed using a combination of malaria-specific supply chains and integrated systems, which further complicates estimates. Supply chain costs in each country also depend on the typology of the system, number of levels in the distribution system, and the types of fixed and variable costs inherent in the structure [14].

In most malaria-endemic countries, access to ACT and RDTs for malaria diagnosis and treatment remains well below targets despite substantial increases in international disbursements to malaria-endemic countries between 2002 and 2012. These increases have slowed in recent years [15,16] and the Global Fund’s new funding model is placing a greater emphasis on value for money [17], making better cost estimates even more important.

The objective of this study was to estimate the supply chain costs for ACT and RDTs from the central to the peripheral levels of the public sector health systems in Benin and Kenya. The two countries were selected to allow for east–west geographical diversity in Africa and to allow for the anomalies in supply chain design in Anglophone and Francophone countries. The countries are also ranked differently on the World Bank logistics performance index (LPI), a composite estimate of road network maturity. Benin has an LPI of 2.85, places 83 out of 155 countries surveyed and is the highest ranked low-income country. Kenya has an LPI of 2.43 and ranks 122 out of the 155 countries. In addition, the SIAPS project presence in both countries facilitated the logistics associated with data collection and follow-up.

The data collected will help countries to better budget and plan for these costs. Specifically the study aimed to accomplish the following:

- Identify the various cost components within the supply chain functions of procurement, storage, transportation, and quality control for ACT and RDTs at each level of the health system, from the central to the peripheral levels.
- Determine the major cost drivers within the supply chain functions.
- Allow accurate estimates to be used for programme planning, budgeting, and policy making decisions.

### Mapping the public sector supply chains in Benin and Kenya for ACT and RDT

Artemether‐lumefantrine (AL) is the first‐line ACT treatment for uncomplicated malaria in Benin and Kenya and, together with RDTs, is procured in both countries using the Global Fund and US President’s Malaria Initiative (PMI) funding. In both countries, supply chains for ACT and RDTs are integrated within the existing public sector supply chain. A mapping of the supply chain structure helped determine how products move from the central to the peripheral level, and identify and estimate associated costs. This was done through a combination of Ministry of Health document reviews and interviews with key stakeholders.

### Benin

**Figure 1:**
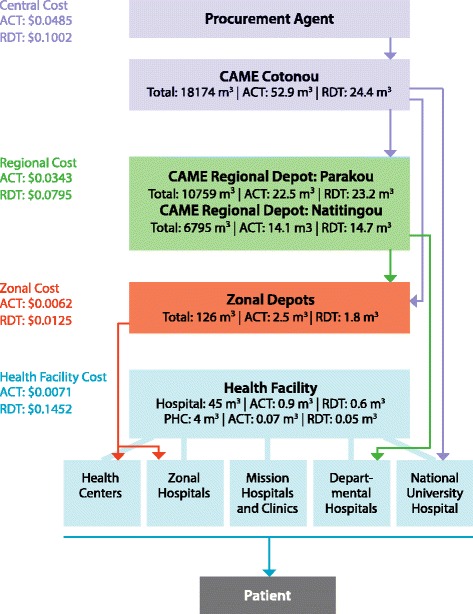
**Supply chain with volumes and costs (2013 USD) in Benin.**

Warehousing and distribution is carried out by *La Centrale d’Achat des Medicaments Essentiels et des Consommables Médicaux* (CAME), an independent, not-for-profit organization (Figure 1). Procurement of ACT and RDTs is done through international procurement agents and coordinated by the *Programme National de Lutte Contre le Paludisme* (PNLP). ACT and RDTs are stored in a dedicated malaria warehouse and are collected by zonal depots or sent to one of two regional depots in Parakou and Natitingou using CAME trucks. Regional stores pick up their stock from the CAME depots and health centres and hospitals collect stock from the regional stores. The national hospital, the *Centre National Hospitalier Universitaire* (CNHU), and departmental hospitals supply themselves directly from CAME Cotonou. Product quality control is carried out by the *Laboratoire National de Contrôle de Qualité* (LNCQ) upon arrival in-country and the costs for this are borne by the public sector.

### Kenya

**Figure 2:**
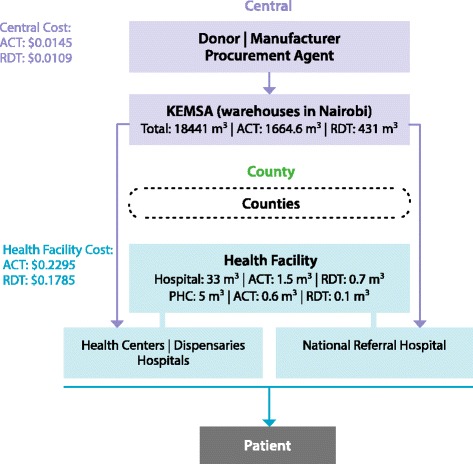
**Supply chain with volumes and costs (2013 USD) in Kenya.**

The Kenya Medical Supplies Agency (KEMSA), a parastatal organization, manages procurement, warehousing, and distribution of medicines and health products in Kenya. It also stores and distributes products procured by other donors directly to health facilities. KEMSA has two warehouses in Nairobi and since 2013, the county governments procure commodities directly either from KEMSA or other suppliers (Figure 2). At the time of this study, however KEMSA was the central procurement agency distributing ACT and RDTs to hospitals, health centres, and dispensaries using a privately contracted transport provider. The costs thus presented in this paper represent this structure. The only quality control costs incurred by the government is post-market surveillance for ACT carried out by the national Pharmacy and Poisons Board (PPB) in collaboration with the National Quality Control Laboratory (NQCL) and the Department of Malaria Control (DoMC).

## Methods

### Costing approach

**Figure 3:**
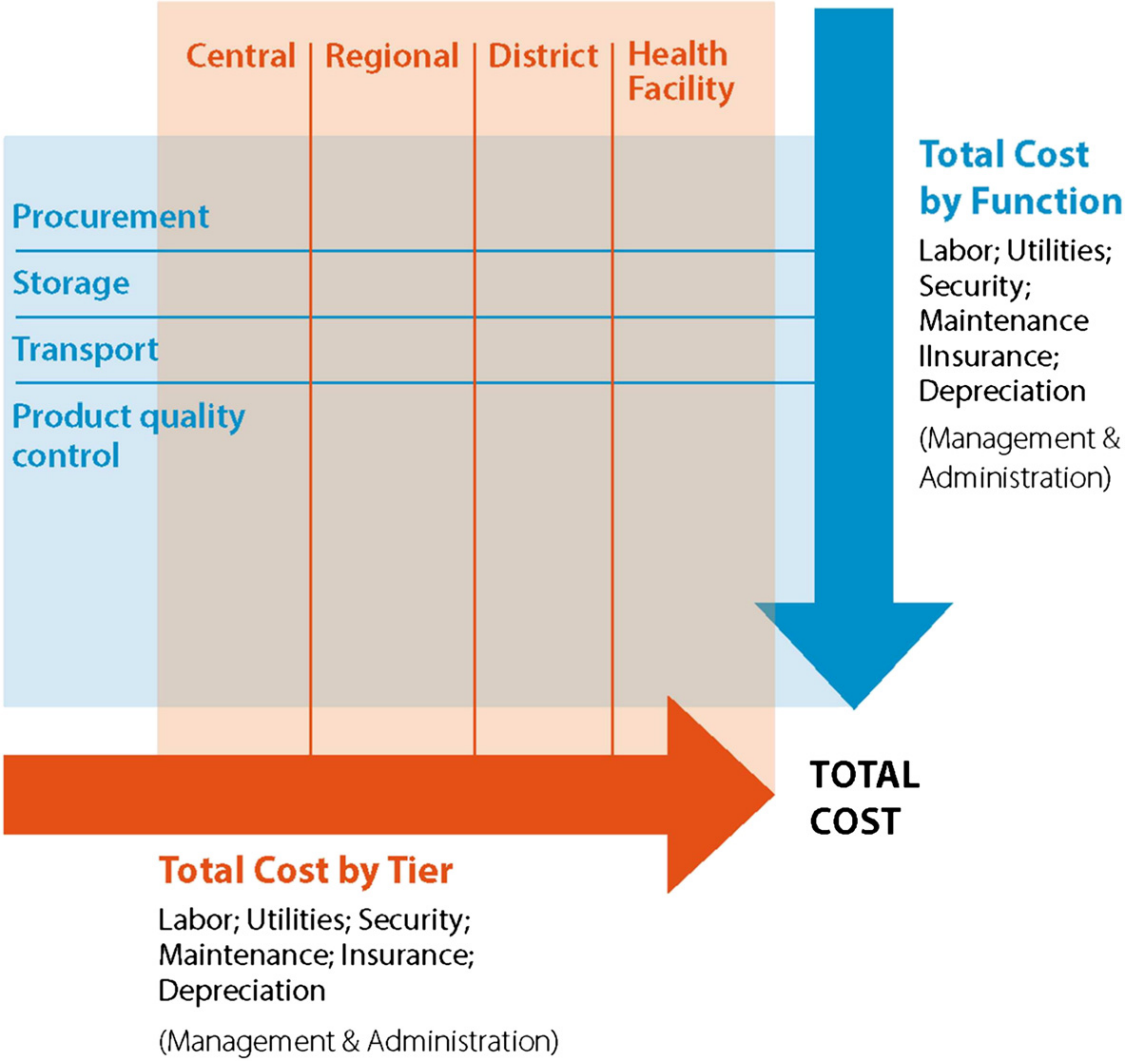
**Supply chain costing overview.**

A micro-costing approach was used which included all fixed and variable costs in the supply chain. Document review and semi-structured interviews were carried out at the central, intermediate, and facility storage levels. Quantitative information came from financial reports, KEMSA and CAME databases, distribution records, and health facility records. Additional information was obtained from logistics reports, transport schedules, expenditure and audit reports. When possible, data on actual rather than budgeted expenditures were used. Extrapolations and inferences were drawn from existing data when detailed information was not available. Labour costs were calculated on the basis of self-reported hours. Figure 3 illustrates the approach used.

An application for ethical approval was made to the University of Michigan Institutional Review Board on July 10, 2013. A determination of “not regulated status” was made (eResearch ID: HUM00078395) and on the basis of this determination, ethical approval was waived by the appropriate bodies in Benin and Kenya.

Survey guides were developed to facilitate collection of data on costs of each supply chain function including procurement, product quality control, storage, and transportation. Cost inputs were identified and included labour, utilities, security, maintenance, insurance and depreciation [8]. All costs are reported in 2013 US dollars (USD).

### Data collection

Data collection took place between September and November 2013. A free, open-source software application, the CommCare® HQ Platform, was used to develop and administer the electronic survey guides to individuals at the central, regional and facility levels and aggregate the data collected.

### Sampling

The sites surveyed were selected from Ministry of Health facility lists. The sampling methodology aimed to reflect geographic variety, malaria endemicity, rural and urban locations, accessibility of health facilities, and other factors that can affect supply chain costs. In addition, sites from each level of the distribution system were surveyed. The methodology was not intended to be statistically representative of the entire country, but rather to isolate the most relevant elements to this costing exercise.

In Benin, 22 health establishments were selected (six distribution depots, five hospitals, and 11 health centres) in three departments of Benin: Atacora, Borgou, and Littoral-Atlantique. Facilities were chosen within a two-hour radius of three city centres: Cotonou, Natitingou, and Parakou. In addition, data were obtained from CAME and PNLP. Specialized health facilities, national hospitals, and provincial facilities which did not carry ACT or RDTs were excluded from the sample. A total of 26 people were interviewed at the central (1), regional (3), depot (6) and facility (16) levels.

In Kenya, 22 health facilities were selected (six hospitals, seven dispensaries, and nine health centres) within 18 km from a main road in three provinces: Coastal, Nairobi, and Nyanza. In addition, data were collected at the three KEMSA warehouses and DoMC. One respondent was interviewed at each of the facilities and two at the PPB and NQCL level. At the KEMSA level, a team of respondents provided the information required for the costing. In both countries respondents were pharmacists, pharmacy technicians, nurses, stock managers or clinicians.

### Annual throughput

Data on the total volume of products moving through the entire supply chain over the course of one year or annual throughput were collected from KEMSA and CAME using shipment history and procurement status reports from July 2012 through June 2013. This included donor procurements. The annual volume and value of products was estimated by multiplying the number of each stock-keeping unit (SKU) by the volume of each unit in cubic metres and the unit price and summing across all products. SKUs of the four ACT packs were included in the analysis (blister packs of 6, 12, 18, and 24 tablets) in addition to RDT kits. In all cases, “ACT” refers to AL in Kenya and Benin.

### Cost categories

Costs for management and administration including any maintenance and information technology (IT) costs were integrated into the functions and not considered as a separate category.

### Procurement costs

Procurement expenses included costs of forecasting, tender development, management, and award. Inputs included labour costs for developing the forecast, bid evaluation, and award as well as costs for advertising tenders in the local media. Annual procurement costs in both Benin and Kenya were calculated by dividing the ACT or RDT tenders by the total number of tenders. The resulting figure was multiplied by the annual cost of the tendering process and divided by the total units of ACT and RDTs, respectively. When products were financed by PMI, they were procured directly through USAID | DELIVER and the government did not incur any procurement cost.

### Product quality control costs

Quality control costs were mainly associated with the collection and testing of products. In Benin, samples from each product batch of ACT were tested upon arrival in the country. LNCQ charges PNLP a fee for testing each batch. Costs were, therefore, calculated according to the number of batches arriving in the country.

In Kenya, the supplier conducts pre-shipment inspection and this cost was, therefore, not included in the estimates. The only costs for quality testing of ACT were from post-marketing surveillance activities carried out at select sites using a Minilab®. The total cost for this activity was readily available from PPB. Secondary testing, when necessary was performed by NQCL which charged a standard price per sample.

### Storage costs

Storage costs included fixed (e.g. infrastructure equipment) and recurrent costs (e.g. utilities, rent, equipment maintenance, stock management, labour, insurance, security, and other administrative expenses). It also included the costs associated with depreciation of equipment, IT, and warehouses and their potential replacement value. Storage costs per unit were calculated according to the total cost figures provided by CAME and KEMSA divided by the total volume distributed that year.

### Transportation costs

Transportation costs included labour costs for staff, depreciation of vehicles, fuel, repairs, maintenance, and insurance. Costs were classified as transportation if the cost was associated with the delivery or pick-up from a storage facility. In Benin, CAME Cotonou made quarterly shipments to its depots in Parakou and Natitingou and staff used health facility vehicles or public transport to obtain commodities from these central tiers. ACT and RDTs were shipped separately and associated costs were easily calculated using the above inputs. In Kenya, transport was subcontracted and these costs were readily available from KEMSA. Shipments were made quarterly and the costs attributable to ACT and RDTs were estimated according to the volume of space occupied in a standard truck.

### Labour costs

Labour costs for supply chain-related tasks were calculated based on self-reported hours during the interview process by staff responsible for managing malaria commodities. Each staff member’s civil service grade was matched to the public service management salary guide to obtain labour costs.

### Data analysis

Our primary objective was to estimate the supply chain costs per unit of ACT or RDT distributed. In all cases a unit was ACT was one treatment course packaged in a single unit pack and a single RDT. This cost was calculated by dividing the annual PSM costs by the annual throughput of all commodities. Throughput was estimated by function, input type, and level of the supply chain. Cost and throughput were expressed in 2013 USD, using an average exchange rate of 494.11 Benin Central African francs and 84.76 Kenyan shillings [18].

The aggregate supply chain cost per unit from the national store to service delivery level was calculated as follows:

Aggregate supply chain cost per unit = [cost per unit at the Central Medical Store (CMS)] + [average cost per dose at intermediate tier(s)] + [average cost per unit for the health centres and hospitals].

Unit costs of ACT and RDTs at health facilities were also calculated. From these, an average cost was derived by taking each facility cost and weighting it to the number of ACT and RDTs procured by that health facility. This cost was then divided by the median $\left(M\overline{X}\right)$ volume procured and multiplied by the volume of a treatment or test (*v*_*ACT*_) to obtain per unit cost for ACT or RDTs. For example, for labour costs per ACT, for every health centre or hospital “i” the formula is represented as follows:

$$ \frac{\Sigma \left( Labor\kern0.5em Cos{t}_i\kern0.5em \times \kern0.5em  Total\kern0.5em  ACT\kern0.5em  Units\kern0.5em  Procure{d}_i\right)/\varSigma \left( Total\kern0.5em  ACT\kern0.5em  Units\kern0.5em  Procure{d}_i\right)}{\left(M\overline{X}\left( Total\kern0.5em  Volume\kern0.5em  Procure{d}_i\right)\right)}\times {v}_{ACT} $$

This calculation was made for each of the other costs: utilities, labour, transportation, security, and depreciation of IT, equipment, and warehouses.

Volumes in cubic metres for each SKU were obtained from KEMSA. Equivalent data were not available in Benin, and therefore KEMSA volumes were used for identical products. For products distributed in Benin that were not stocked by KEMSA, standard volume data for products from the USAID | DELIVER project were used [19]. When data were not otherwise available, proxy volumes from products with similar volumes were used.

The replacement costs for the government-owned storage facilities, associated buildings, and fixed assets (e.g., vehicles and equipment) were estimated using straight-line depreciation. Staff and the portion of other resources allocated specifically to ACT and RDTs were identified. Where this information was not available, weighted averages were used to apportion the costs specifically to these products.

Most health facilities perform mainly clinical activities; therefore the administrative function of a facility covered several other non–logistics-related functions. The cost of administrative supply chain activities at the health facility level was estimated to be 10% of total administrative operating costs. For clinics, 50% of operating costs were apportioned to storage activities. In Benin, zonal depots were dedicated to storage activities and, therefore, incurred 100% of operating costs. Similarly, CAME warehouses had spaces dedicated to anti-malarials and hence also incurred 100% of the related expenditures.

The various cost drivers in each of the four functions were assessed to determine if one function accounted for a large proportion of costs relative to the others, and how changes in the quantity or volume of goods passing through the system affected each function. Depending on the nature of the supply chain function, either volume-proportional or value-proportional cost allocations were used. In the volume-proportional cost allocation method, the fixed costs and overhead costs were allocated to the products in proportion to the relative volume in quantity or cubic metre. In the value-proportional cost allocation method, the fixed costs and overhead costs were allocated to malaria commodities in proportion to the monetary value of the products. Volume-proportional cost allocation, as measured by the quantity of product, was used to allocate costs related to procurement (tender management) and quality assurance. For example, each ACT unit incurs a cost for quality assurance activities. To determine this per unit cost, the total yearly cost of testing ACT was divided by the total number of units shipped from KEMSA for that same year normalized to USD1. Storage and transport functions were analyzed using volume-proportional cost allocation in cubic metres. Value-proportional cost allocation was used only to estimate insurance costs.

Given the uncertainty in the cost estimates, the standard deviation (SD) from the mean weighted costs was calculated [see Additional files 1 and 2].

## Results

### Annual throughput

In Kenya, KEMSA delivered a total volume of 18,441 cubic metres of ACT and RDTs from July 2012 to June 2013 including 14,979,359 treatments of AL and 7,277,960 individual RDT tests. A median of 1,827 treatment courses of ACT was distributed to each facility, which made up 9% of total volume distributed by KEMSA. RDTs represented 2% of total volume of product distributed. The total value distributed was USD 17,292,353 of ACT and USD 5,153,328 of RDTs (calculated from PMI landed cost per RDT and Global Fund price quality reporting cost per treatment).

In Benin, the exact volumes of commodities were unknown at the lower tiers - a value required to calculate supply chain cost by tier hence, simple heuristics were used to develop estimates of product flow volume as follows. Using data provided by CAME on the total quantity of pharmaceuticals and anti-malarials shipped to the regional warehouses and zonal depots and knowledge of the supply chain network illustrated in Figure 1, it was determined that depots represented 12% of the demand for CAME’s general pharmaceutical products and 94% and 98% for ACT and RDTs, respectively. Among the depots, ACT represented 0.7% of total volume and RDTs represented 0.02% of total volume procured from CAME Cotonou. To determine the total volume procured from the CAME warehouses in Parakou and Natitingou, the average proportion of ACT and RDTs to total volume of all commodities was used. Using the number of health centres and hospitals serviced by each province and known demand data from a hospital and health centre in Cotonou, it was determined that 30% of depot volume went to hospitals and 70% of volume was split across other health centres. The total value of ACT distributed between July 2012 and June 2013 was USD 377,258; the total value for RDTs was USD 224,800. These were based on the unit value calculated from PMI total acquisition costs multiplied by quantity of products distributed.

Figures 1 and 2 illustrate the throughput and unit costs within the supply chain structure in Benin and Kenya. In each chart, the volumes represent the distribution flow to each storage facility over the course of one year. Calculations were made according to the average volumes distributed to the facilities from the sample. Values of ACT and RDT are the distribution costs for each tier per unit in USD (2013) normalized to USD 1.

### Total supply chain costs

Total costs were analyzed by level, function, and cost category. This section summarizes the cost and cost drivers, and determines how sensitive distribution costs are to changes in a product’s acquisition cost.

### Central-level costs

The main central-level procurement costs incurred were for tender management and advertising. In both Benin and Kenya, the national quality control laboratories charged the malaria programmes for each batch tested.

Total costs for tendering in Benin amounted to USD 2,819 for ACT for 2012–2013. RDTs were procured through PMI and, therefore, incurred no procurement charges to the government. USD 160 was charged per batch of ACT and USD 200 per batch of RDTs for a total annual cost of USD 648 and USD 2,024 for quality testing of ACT and RDTs, respectively.

In Kenya, procurement costs for tender management amounted to USD 13,998 for ACT and USD 4,666 for RDTs. and the total cost for post-marketing surveillance of ACT in the public sector was USD 3,749. RDTs were not tested and, therefore, did not incur a cost.

### Central storage and distribution level costs

In Benin, central-level storage and distribution costs for ACT and RDTs from CAME Cotonou were estimated at a total of USD 26,163. To transport ACT and RDTs along with antiretrovirals to regional warehouses in Parakou and Natitingou, the cost was USD 1,821 per trip. Four trips were made during the assessment year, and ACT and RDTs made up an estimated two-thirds of the volume transported for a total annual transportation cost of USD 4,857. Regional depots incurred an annual cost of USD 21,014 to store ACT and RDTs. At KEMSA, central-level management costs for ACT and RDTs were USD 249,675. The annual cost to transport ACT and RDTs by a third-party contractor directly to health facilities was USD 440,210.

### Peripheral-level storage and distribution costs

In Benin, the median annual operating cost across depots was USD 8,394, across hospitals was USD 14,788, and across clinics was USD 4,755. In Kenya, the median operating cost for annual storage of all commodities was USD 15,288 at hospitals and USD 13,054 at clinics. These estimates were obtained by multiplying the unit cost by the total quantity distributed.

### Supply chain costs per unit of ACT and RDT distributed

Using data from each distribution tier, the total supply chain costs of ACT and RDTs were estimated. Prices were normalized to USD 1 of product flowing through the distribution channel for simple comparison.

**Figure 4:**
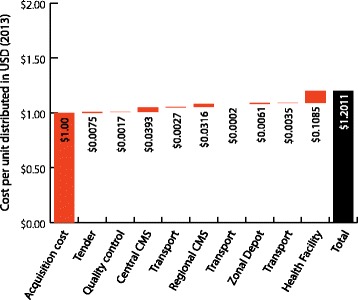
**ACT cost breakdown normalized to USD 1 (2013) in Benin.**

**Figure 5:**
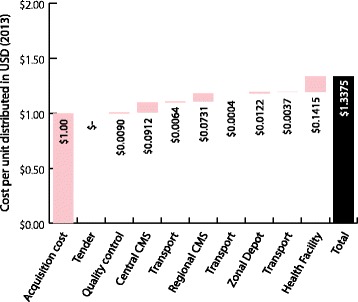
**RDT cost breakdown normalized to USD 1 (2013) in Benin.**

**Figure 6:**
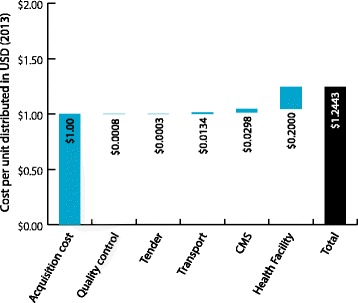
**RDT cost breakdown normalized to USD1 (2013) in Kenya.**

**Figure 7:**
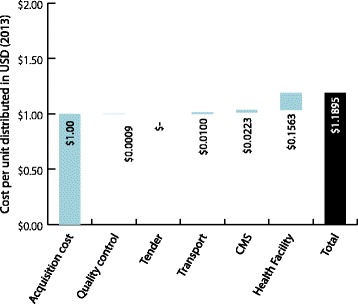
**RDT cost breakdown normalized to USD 1 (2013) in Kenya.**

Tables 1, 2, 3 and 4 and Figures 4, 5, 6, and 7 illustrate the cumulative supply chain costs for distribution of ACT and RDTs from the central level to the peripheral level normalized to USD 1. In Benin, supply chain costs added USD 0.2011 (SD = 0.1216) to the initial acquisition cost of ACT and USD 0.3375 (SD = 0.1544) to the cost of RDTs.

**Table 1:** **Unit costs for ACT in Benin**

	**Central level**	**CMS**	**Regional warehouse**	**Zonal store**	**All health facilities**	**Total**
**Product quality testing**	$ 0.0017	$ -	$ -	$ -	$ -	$ 0.0017
**Announcements in local media**	$ 0.0005	$ -	$ -	$ -	$ -	$ 0.0005
**Insurance for commodities**	$ -	$ 0.0000	$ -	$ -	$ -	$ 0.0000
**Utilities**	$ -	$ 0.0008	$ 0.0004	$ 0.0016	$ 0.0472	$ 0.0501
**Labour**	$ 0.0069	$ 0.0369	$ 0.0298	$ 0.0024	$ 0.0386	$ 0.1147
**Security**	$ -	$ 0.0001	$ 0.0006	$ 0.0001	$ 0.0080	$ 0.0089
**Other SG & A**	$ -	$ 0.0000	$ 0.0000	$ -	$ -	$ 0.0000
**Maintenance**	$ -	$ 0.0001	$ 0.0001	$ -	$ -	$ 0.0002
**Rent - Warehouse**	$ -	$ -	$ -	$ -	$ -	$ -
**Depreciation**	$ -	$ 0.0013	$ 0.0006	$ 0.0019	$ 0.0145	$ 0.0184
**Transport**	$ -	$ -	$ 0.0027	$ 0.0002	$ 0.0037	$ 0.0066
**Total**	$ 0.0092	$ 0.0393	$ 0.0343	$ 0.0062	$ 0.1120	$ 0.2011

**Table 2:** **Unit costs for RDTs in Benin**

	**Central level**	**CMS**	**Regional warehouse**	**Zonal store**	**All health facilities**	**Total**
**Product quality testing**	$ 0.0090	$ -	$ -	$ -	$ -	$ 0.0090
**Announcements in local media**	$ -	$ -	$ -	$ -	$ -	$ -
**Insurance for commodities**	$ -	$ 0.0000	$ -	$ -	$ -	$ 0.0000
**Utilities**	$ -	$ 0.0019	$ 0.0010	$ 0.0036	$ 0.0568	$ 0.0634
**Labour**	$ -	$ 0.0856	$ 0.0692	$ 0.0056	$ 0.0521	$ 0.2125
**Security**	$ -	$ 0.0003	$ 0.0015	$ 0.0003	$ 0.0111	$ 0.0132
**Other SG & A**	$ -	$ 0.0001	$ 0.0000	$ -	$ -	$ 0.0001
**Maintenance**	$ -	$ 0.0003	$ 0.0002	$ -	$ -	$ 0.0005
**Rent - Warehouse**	$ -	$ -	$ -	$ -	$ -	$ -
**Depreciation**	$ -	$ 0.0030	$ 0.0014	$ 0.0025	$ 0.0214	$ 0.0283
**Transport**	$ -	$ -	$ 0.0063	$ 0.0004	$ 0.0038	$ 0.0104
**Total**	$ 0.0090	$ 0.0912	$ 0.0795	$ 0.0125	$ 0.1453	$ 0.3375

**Table 3:** **Unit costs for in Kenya**

**Cost categories**	**Central level**	**CMS**	**All health facilities**	**Total**
**Product quality testing**	$ 0.0003	$ -	$ -	$ 0.0003
**Announcements in local Media**	$ 0.0002	$ -	$ -	$ 0.0002
**Insurance for commodities**	$ -	$ 0.0000	$ -	$ 0.0000
**Utilities**	$ -	$ 0.0011	$ 0.0057	$ 0.0067
**Labour**	$ 0.0006	$ 0.0103	$ 0.1615	$ 0.1724
**Security**	$ -	$ 0.0001	$ 0.0132	$ 0.0133
**Other SG & A**	$ -	$ 0.0010	$ -	$ 0.0010
**Maintenance (IT and equipment)**	$ -	$ 0.0004	$ 0.0010	$ 0.0014
**Rent - Warehouse**	$ -	$ 0.0001	$ -	$ 0.0001
**Depreciation**	$ -	$ 0.0006	$ 0.0186	$ 0.0192
**Transport**	$ -	$ -	$ 0.0298	$ 0.0298
**Total**	$ 0.0011	$ 0.0134	$ 0.2298	$ 0.2443

**Table 4:** **Unit costs for RDTs in Kenya**

**Product quality testing**	$ -	$ -	$ -	$ -
**Announcements in local Media**	$ 0.0002	$ -	$ -	$ 0.0002
**Insurance for commodities**	$ -	$ 0.0000	$ -	$ 0.0000
**Utilities**	$ -	$ 0.0008	$ 0.0058	$ 0.0066
**Labour**	$ 0.0007	$ 0.0077	$ 0.1222	$ 0.1306
**Security**	$ -	$ 0.0000	$ 0.0103	$ 0.0103
**Other SG & A**	$ -	$ 0.0007	$ -	$ 0.0007
**Maintenance (IT and equipment)**	$ -	$ 0.0003	$ 0.0003	$ 0.0005
**Rent - Warehouse**	$ -	$ 0.0001	$ -	$ 0.0001
**Depreciation**	$ -	$ 0.0004	$ 0.0177	$ 0.0181
**Transport**	$ -	$ -	$ 0.0223	$ 0.0223
**Total**	$ 0.0009	$ 0.0100	$ 0.1785	$ 0.1895

In Kenya, supply chain costs added USD 0.2443 (SD = 0.0183) to the acquisition cost of ACT and USD 0.1895 (SD = 0.0471) for RDTs. When these values were calculated as percentages of the landed cost of ACT and RDTs in the respective countries, the percentage values obtained were 20% for ACT and 34% for RDTs in Benin and 24% for ACT and 19% for RDTs in Kenya. In both countries and across both products, the highest costs were incurred during the “last mile” of distribution at the health facility. Across the functions, labour, transportation and utilities incurred the highest costs, particularly at the health facility level.

**Figure 8:**
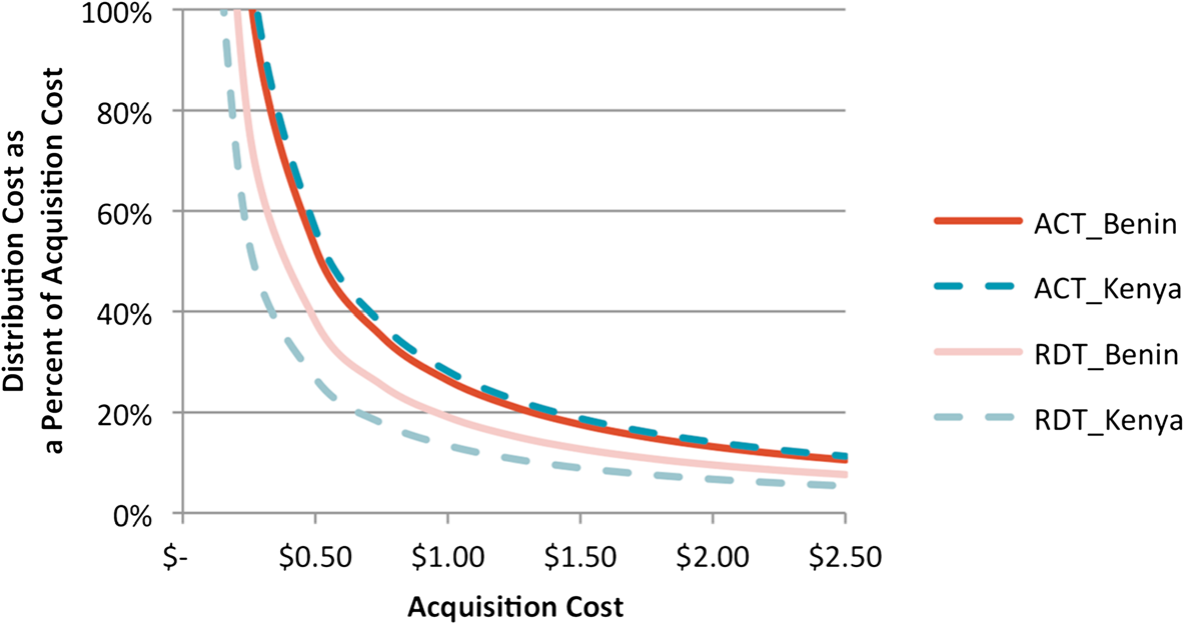
**Sensitivity analysis on distribution cost as a percent of acquisition cost.**

A sensitivity analysis performed on the acquisition costs of ACT and RDTs in both countries against the percentage contribution of the supply chain (Figure 8) illustrated the volatility and inaccuracy of estimating the distribution cost as a percentage of the initial acquisition cost. This high sensitivity was due to the association of over 99% of costs to the volume of the product distributed. The only distribution cost directly dependent on product value was commodity insurance which was low relative to other costs.

### Disaggregating hospitals and health centres and urban and rural facilities

Hospital and health facility costs were disaggregated by estimating the respective costs and weighting them to the total number of each facility type. The resulting estimate was a lower per unit of product than when hospitals and health centres were aggregated. In Benin, the resulting distribution cost was 14% of acquisition cost for ACT and 26% for RDTs and 13% and 10% for ACT and RDTs in Kenya. In both Benin and Kenya, the per-unit cost of storage at health clinics was much higher than at hospitals. Even though the overall operating costs of a hospital were higher in Kenya, the per unit cost for storing ACT at health clinics was approximately six times higher than at hospitals due to the lower volume of products stored at health centre level. Similarly, storage costs for RDTs were three times higher at health clinics than those incurred at hospitals.

Additionally, costs by rural and urban facilities were also disaggregated. Operating costs were approximately 1.5 times higher in rural facilities than urban ones while transport costs were up to 40% higher in rural facilities. Furthermore, the volume procured by urban facilities was five times greater than rural facilities with 30% more ACT and RDTs than rural facilities. In both countries, disaggregating the urban and rural facilities decreased the supply chain cost estimate in urban facilities to 10% and 23% in Benin and 23 and 19% in Kenya for ACT and RDTs. A smaller difference was obtained between urban and rural facilities in Benin.

### Major cost drivers

**Figure 9:**
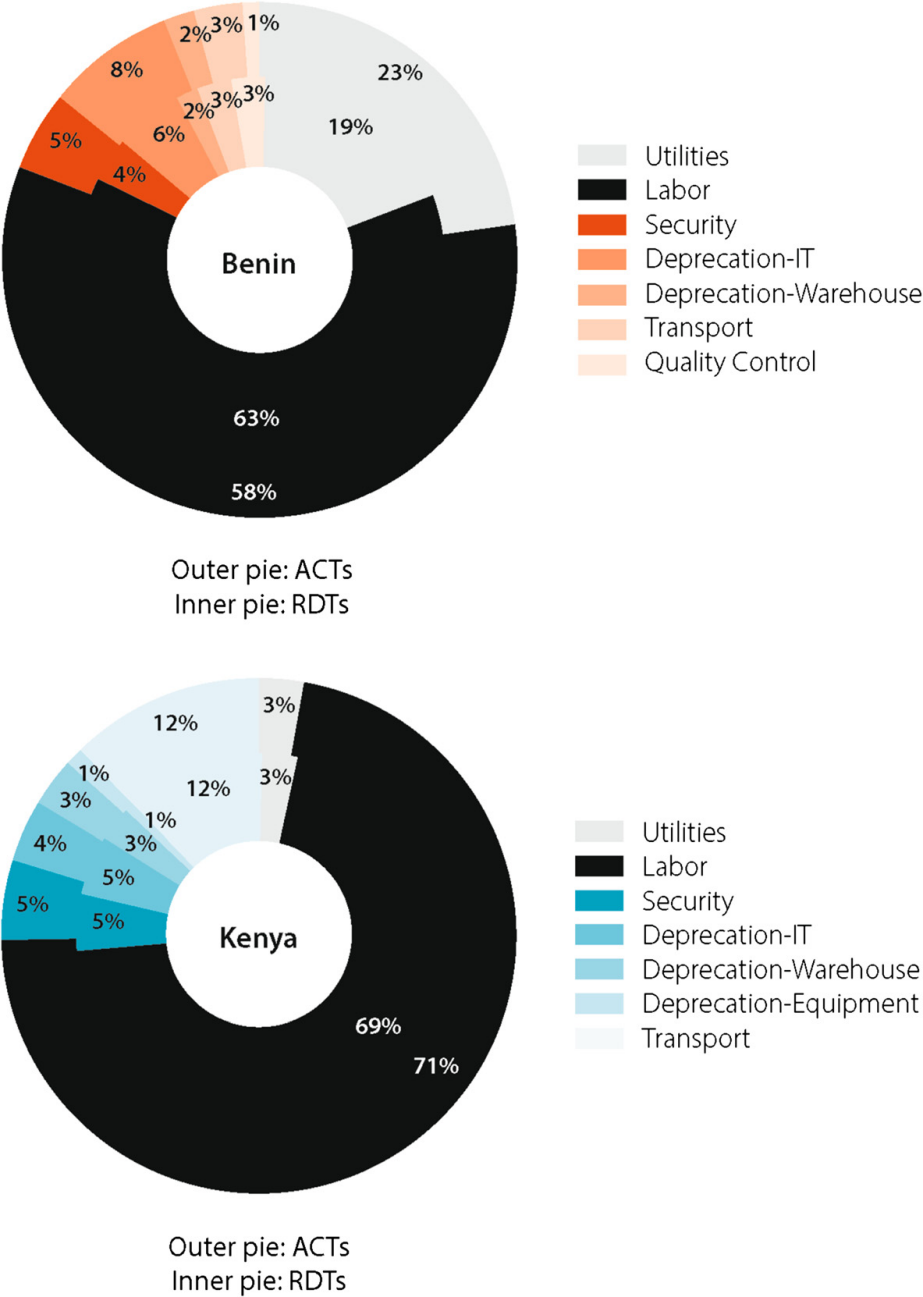
**Breakdown of cost drivers for ACT and RDTs in Benin and Kenya.**

The median annual labour cost for depots was USD 3,155, USD 3,226 for hospitals, and USD 1,918 for clinics. Labour was the highest cost contributor at all levels accounting for on average 62% of total supply chain costs. Although salaries themselves are not unreasonably high, the high labour cost can largely be attributed to large number of employees or potential lower productivity. Utilities were the second largest cost driver, accounting for about 20% of cost followed by transportation at 12% of cost. The primary utility cost was electricity, and the primary cost driver for transport was fuel, which amounted to over 20% of total transportation cost. Utility costs were higher in Benin than Kenya whereas the reverse was true for labour costs. Thus, proportionally, utility costs appear to be significantly more in Benin. Figure 9 illustrates the cost drivers for ACT and RDTs in Benin and Kenya.

## Discussion

This study identified the structural and operational supply chain costs associated with distribution of ACT and RDTs in Benin and Kenya. The cost of procurement, distribution, and product quality control was estimated using an output metric of cost per USD value of an ACT and RDT unit distributed. Other studies have used a variety of cost metrics: costs per USD value [20-22]; costs per kilogram [22], and costs per kilometre [20-22]. These studies estimated costs to be USD 0.63–1.15 for vaccines in Mozambique [21], USD 0.24–2.00 for general products in Nigeria [20]; and 7.6–24% for antiretrovirals, essential medicines and vaccines in Zambia, Uganda and Niger, respectively [23-25].

The findings indicate that supply chain costs play a significant role in the total cost of delivering ACT and RDTs. A few cost drivers stand out in both Benin and Kenya - labour, transport, and utilities. At the level of storage, labour and utilities stand-out accounting for up to 71% of the total distribution costs of ACT in Kenya.

It should be noted that labour cost estimates for ACT and RDT stock-management were based on self-reported hours during interviews with heath workers, a process likely to be associated with reporting bias. However, despite this, these findings are consistent with those from other studies [22,23,25], although in the case of vaccines, high cold chain and equipment costs make total supply chain costs higher and, therefore, labour becomes a smaller percentage of total supply chain costs (Lydon et al. 2014 [3]). In addition, labour costs may be co-funded by the countries themselves and often not included in proposals to donors. The costs in this paper, however, represent the total resources that countries are dedicating to distribution.

The analysis also established that supply chain costs per ACT unit varied by the level of the system with costs increasing as the product reaches the last mile. In both countries, despite having very different health system structures, the costs tended to be the highest at the level of the health centre or dispensary in comparison to the central or intermediate storage depots. A further breakdown of the health facilities by hospitals and primary health clinics illustrated that health clinics were much more expensive than hospitals for supply chain functions per unit of product. Although the overall operating costs of a hospital were higher, clinic storage costs were approximately six times the cost of storage at hospitals for ACT and three times for RDTs mostly due to the higher volume of ACT and RDTs flowing through hospitals. These findings are consistent with those from other studies [7,21,25,26]. Central storage costs for anti-malarials were much higher in Benin possibly due to the use of dedicated warehouses and labour for their management. Similarly in Kenya, operating costs were found to be about three times higher for ACT and RDTs in rural facilities than urban ones. The total volume procured by urban facilities was five times greater than that procured by rural facilities and higher transport costs to rural facilities may play a part in this observation.

The findings also illustrated the importance of product volumes as measured by product dimensions in estimating costs of the supply chain. A greater portion of the storage costs are fixed and reliant on volume; therefore, when considering the costs attributed to a unit of product, as volume increases, costs proportionately decrease. In the case of quality control, costs are often a rate attached to the batch size tested. Cost per unit of product will therefore stay relatively stable despite fluctuation in total volume. The study provided evidence that using a percentage of the acquisition costs to estimate supply chain costs can be inaccurate. This has major implications for the way costs for procurement and SCM are currently estimated. These findings indicate that using “ballpark” percentages to estimate supply chain costs, may cause shortfalls in resource allocations for supply chain functions. Countries should be supported to generate more accurate estimates in the future.

The authors note that the costs described in this study are for the existing public sector supply system, including current inefficiencies [27-30]. This work does not separate inefficiencies but rather builds them into the cost analysis. The study does not identify such inefficiencies in the system, create an optimization model or suggest what an ideally operating system should cost. Doing so would require additional data and analysis on service-level indicators including product availability, quality, and price. While this work moved costing closer toward transparency and accuracy, it is only a glimpse of supply chain operations Kenya and Benin. A more complete picture needs routine data collection with sufficient time-lapsed data points to allow statistical conclusions to be made. In addition to greater cost transparency, this could lead to a richer understanding of operational needs. This cost data however provides valuable information for these additional analyses including information for economic evaluations.

This study has a few limitations. Although the proportional cost of the supply chain was obtained in this research, it is specific to the ACT and RDTs in distributed in Benin and Kenya. These percentages are not to be applied generally by a CMS across all the products of varying value and volume. Two additional considerations that should be made are time allocation, which is the driving force behind labour cost, and specific distribution requirements, such as those for a cold chain. Secondly, the sampling methodology was not intended to be statistically representative of the entire country but was rather directed at identifying the key variables that may influence supply chain cost which may have introduced a level of selection bias.

While an accurate costing of the supply chain is important for budgeting and planning, conducting a micro-costing exercise is a highly resource- and time-intensive effort. Given that the supply chain costs vary according to the acquisition cost, a better methodology is needed for estimating costs rather than using a fixed percentage. Three options should be considered in place; one option is to use different weights for the costs of the supply chain for high and low-value products. A second option would be to use algorithms of different handling fees based on budget line items [10], which would entail separating products and allocating different proportions to each group. Lastly, the authors recommend using a simple extrapolative model to estimate distribution costs at country level. This remains a priority for researchers and implementers working in this arena.

## Conclusion

Unpacking the supply chain costs has several advantages. First, countries and donors can identify the major cost drivers where a more in-depth analysis may be required to identify opportunities for improved efficiency particularly if major discrepancies between similar countries are found. Second, it allows donors and countries to separate the costs for the supply chain tiers and functions and allows identifying areas of support based on an empirical evaluation of costs. Third, it allows countries to demonstrate how government funds are being leveraged for procurement and SCM functions in proposals. The study provided information that can be used by policymakers to understand the costs of the supply chain for ACT and RDTs and to advocate for the efficient allocation of resources for future programme needs.

In conclusion, product volumes should be considered when costing distribution rather than value. Countries should avoid using percentages of total acquisition costs to estimate the cost of product distribution, and planners and policy makers can use the information on cost drivers to conduct a more detailed analysis to determine how to increase efficiency. Given that countries should use accurate distribution cost data when budgeting for malaria interventions and that costing studies are expensive and time-intensive, a model to generate more accurate estimates in the future, by estimating costs according to volume rather than value would be embraced by stakeholders. Furthermore, additional country-level studies are needed to capture country-specific costs and to draw inferences across different supply chain structures.

## Additional files

Additional file 1:
**Uncertainty analysis for Benin (Costs in USD 2013 Normalized to USD 1).**
Additional file 2:
**Uncertainty analysis for Kenya (Costs in USD 2013 Normalized to USD 1).**

## Abbreviations

ACT: Artemisinin-based combination therapy
AL: Artemether-lumefantrine
CAME: *La Centrale d'Achat des Médicaments Essentiels et Consommables Médicaux*
CNHU: *Centre National Hospitalier Universitaire*
CMS: Central Medical Store
DoMC: Department of Malaria Control
Global Fund: The Global Fund to Fight AIDS, Tuberculosis and Malaria
IT: Information technology
KEMSA: Kenya Medical Supplies Agency
LNCQ: *Laboratoire National de Contrôle de Qualité*
LPI: Logistics Performance Index
MDG: Millennium Development Goals
MSH: Management Sciences for Health
NQCL: National Quality Control Laboratory
PMI: (United States) President’s Malaria Initiative
PNLP: *Programme National de Lutte contre le Paludisme*
PPB: Pharmacy and Poisons Board
PSM: Procurement and supply chain management
RDT: Rapid diagnostic test
SCM: Supply chain management
SD: Standard deviation
SIAPS: Systems for Improved Access to Pharmaceuticals and Services Programme
SKU: Stock-keeping unit
USAID: US Agency for International Development
USD: United States dollar
WDI: William Davidson Institute
