# Supplementary material for: Costing the supply chain for delivery of ACT and RDTs in the public sector in Benin and Kenya
Source: Malar J. 2015 Feb 5;14:57. doi: 10.1186/s12936-014-0530-1 (PMC4341244; doi:10.1186/s12936-014-0530-1)
Supplement: Additional file 1: — Uncertainty analysis for Benin (Costs in USD 2013 Normalized to USD 1). [file 12936_2014_530_MOESM1_ESM.docx]

**Additional file 1: Uncertainty Analysis for Benin (Costs in USD 2013 Normalized to USD 1)**

ACT

RDT

Weighted Average

Weighted Standard Deviation

Weighted Average

Weighted Standard Deviation

LNCQ

Post-shipment Testing

n = 1: statistical tests not applicable

Post-market Testing

PNLP

Announcements in Local Media

n = 1: statistical tests not applicable

Labor

CAME
Siège

Insurance for commodities

n = 1: statistical tests not applicable

Utilities

Labor

Security

Other SG&A

Maintenance - IT

Maintenance - Equipment

Rent - Warehouse

Depreciation - IT

Depreciation - Equipment

Depreciation - Warehouse

Transportation

CAME
Dépot

Insurance for commodities

n = 2: statistical tests not applicable

Utilities

Labor

Security

Other SG&A

Maintenance - IT

Maintenance - Equipment

Rent - Warehouse

Depreciation - IT

Depreciation - Equipment

Depreciation - Warehouse

Transportation

$ 0.0002

$ 0.0002

$ 0.0002

$ 0.0001

Dépot repartiteur de zone sanitaire (sample of 6 facilities)

Insurance

$ -

$ -

$ -

$ -

Utilities

$ 0.0016

$ 0.0012

$ 0.0012

$ 0.0006

Labor

$ 0.0024

$ 0.0008

$ 0.0028

$ 0.0006

Security

$ 0.0001

$ 0.0001

$ 0.0001

$ 0.0001

Maintenance - IT

$ -

$ -

$ -

$ -

Maintenance - Equipment

$ -

$ -

$ -

$ -

Depreciation - IT

$ 0.0015

$ -

$ 0.0019

$ -

Depreciation - Equipment

$ 0.0001

$ 0.0001

$ 0.0001

$ 0.0000

Depreciation - Warehouse

$ 0.0003

$ 0.0004

$ 0.0005

$ 0.0003

Transportation - all 4 tiers

$ 0.0028

$ 0.0043

$ 0.0091

$ 0.0034

Hôpital de zone sanitaire & Centre de santé (sample of 16 facilities)

Insurance

$ -

$ -

$ -

$ -

Utilities

$ 0.0472

$ 0.0431

$ 0.0568

$ 0.0603

Labor

$ 0.0488

$ 0.0240

$ 0.0675

$ 0.0366

Security

$ 0.0166

$ 0.0203

$ 0.0617

$ 0.0232

Maintenance - IT

$ -

$ -

$ -

$ -

Maintenance - Equipment

$ -

$ -

$ -

$ -

Depreciation - IT

$ 0.0165

$ 0.0206

$ 0.0479

$ 0.0218

Depreciation - Equipment

$ 0.0015

$ 0.0018

$ 0.0052

$ 0.0024

Depreciation - Warehouse

$ 0.0006

$ 0.0048

$ 0.0016

$ 0.0049

Total

$ 0.1401

$ 0.1216

$ 0.2566

$ 0.1544
