# Supplementary material for: Costing the supply chain for delivery of ACT and RDTs in the public sector in Benin and Kenya
Source: Malar J. 2015 Feb 5;14:57. doi: 10.1186/s12936-014-0530-1 (PMC4341244; doi:10.1186/s12936-014-0530-1)
Supplement: Additional file 2: — Uncertainty analysis for Kenya (Costs in USD 2013 Normalized to USD 1). [file 12936_2014_530_MOESM2_ESM.docx]

**Additional file 2: Uncertainty Analysis for Kenya (Costs in USD 2013 Normalized to USD 1)**

ACT

RDT

Weighted Average

Weighted Standard Deviation

Weighted Average

Weighted Standard Deviation

PPB

Procurement Costs

n = 1: statistical tests not applicable

Transport

Labor

Other SG&A

Asset Depreciation

NQCL

ACT Testing

n = 1: statistical tests not applicable

KEMSA

Tender

n = 1: statistical tests not applicable

Insurance for commodities

Utilities

Labor

Security

Other SG&A

Maintenance - IT

Maintenance - Equipment

Rent - Warehouse

Depreciation - IT

Depreciation - Equipment

Depreciation - Warehouse

Transporter payments

(sub) District Hospital, Health Centre, & Dispensary (sample of 21 facilities)

Insurance

$ -

$ -

$ -

$ -

Utilities

$ 0.0010

$ 0.0010

$ 0.0003

$ 0.0003

Labor

$ 0.0289

$ 0.0102

$ 0.0712

$ 0.0373

Security

$ 0.0024

$ 0.0017

$ 0.0023

$ 0.0006

Maintenance - IT

$ 0.0001

$ 0.0001

$ 0.0001

$ 0.0001

Maintenance - Equipment

$ 0.0002

$ 0.0005

$ 0.0003

$ 0.0003

Depreciation - IT

$ 0.0017

$ 0.0017

$ 0.0024

$ 0.0027

Depreciation - Equipment

$ 0.0003

$ 0.0003

$ 0.0002

$ 0.0001

Depreciation - Warehouse

$ 0.0011

$ 0.0028

$ 0.0041

$ 0.0057

Total

$ 0.0358

$ 0.0183

$ 0.0809

$ 0.0471
